# Cell-to-Cell Communication Circuits: Quantitative Analysis of Synthetic Logic Gates

**DOI:** 10.3389/fphys.2012.00287

**Published:** 2012-07-25

**Authors:** Marta Hoffman-Sommer, Adriana Supady, Edda Klipp

**Affiliations:** ^1^Theoretical Biophysics, Institute of Biology, Humboldt-Universität zu BerlinBerlin, Germany

**Keywords:** synthetic biology, mathematical model, yeast, pheromone pathway, HOG pathway

## Abstract

One of the goals in the field of synthetic biology is the construction of cellular computation devices that could function in a manner similar to electronic circuits. To this end, attempts are made to create biological systems that function as logic gates. In this work we present a theoretical quantitative analysis of a synthetic cellular logic-gates system, which has been implemented in cells of the yeast *Saccharomyces cerevisiae* (Regot et al., 2011). It exploits endogenous MAP kinase signaling pathways. The novelty of the system lies in the compartmentalization of the circuit where all basic logic gates are implemented in independent single cells that can then be cultured together to perform complex logic functions. We have constructed kinetic models of the multicellular IDENTITY, NOT, OR, and IMPLIES logic gates, using both deterministic and stochastic frameworks. All necessary model parameters are taken from literature or estimated based on published kinetic data, in such a way that the resulting models correctly capture important dynamic features of the included mitogen-activated protein kinase pathways. We analyze the models in terms of parameter sensitivity and we discuss possible ways of optimizing the system, e.g., by tuning the culture density. We apply a stochastic modeling approach, which simulates the behavior of whole populations of cells and allows us to investigate the noise generated in the system; we find that the gene expression units are the major sources of noise. Finally, the model is used for the design of system modifications: we show how the current system could be transformed to operate on three discrete values.

## Introduction

Mathematical modeling in cellular biology aims to predict the behavior of biological systems. This aspect is particularly important in synthetic biology, where novel biological entities are being designed and implemented. Often, only an intuitive, qualitative prediction is available of how the planned device will function – while quantitative characterization is performed after experimental implementation. Ideally, mathematical modeling should offer the advantage of predicting the quantitative behavior of the system, minimizing the necessity for experimental optimization and fine-tuning. In this work, we present a kinetic model of an experimentally implemented, functioning synthetic system and we show that our model allows for analysis and prediction of the timing of events in the system and facilitates system optimization. Finally, we demonstrate how the model aids the design of system modifications.

For our analysis we used the synthetic cellular logic-gates system developed by Regot et al. (2011), who implemented their system in cells of the yeast *Saccharomyces cerevisiae*, exploiting endogenous Mitogen-Activated Protein Kinase (MAPK) signaling pathways. Such logic-gate units are intended as a first step on the way to the construction of a biological computation device. The novelty of this system lies in the concept of using communicating populations of cells: in each cell type, only a single logic operation is implemented, but the different cell types communicate with each other by secreting diffusible signaling molecules, such that more complicated functions can be achieved by mixing of cells that perform different basic operations. The advantage of this kind of implementation is that the chemical output synthesized by a population of engineered cells – the concentration of the signaling molecule that “wires” the different cell types into a functional circuit – is robust to noise arising in single cells, since it is averaged over the whole population (Li and You, 2011). It also enables the construction of many different functions from a limited number of engineered cells, by just mixing them in different combinations. The attractiveness of this system prompted us to analyze it deeper and to explore the possibilities of its further development.

In the system studied in this work, cell-to-cell communication is based on one of the best studied cell communication systems, i.e., the mating response of haploid yeast cells (Elion, 2000). In the mating process, *MAT*α cells produce the α-factor mating pheromone and express an alpha-factor receptor while *MAT* alpha cells produce alpha-factor and an α-factor receptor. Binding of the cognate pheromone to its receptor stimulates a G-protein-coupled sensing device including a MAPK cascade, which in turn initiates a cascade of events that lead to mating and cell fusion (Dohlman and Thorner, 2001; Hohmann, 2002). Another extensively studied signaling pathway used here is the High Osmolarity Glycerol (HOG) MAPK signaling network (de Nadal et al., 2002; Hohmann, 2002). Several mathematical models of the pheromone-response pathway and the HOG signaling pathway have been already published (Kofahl and Klipp, 2004; Klipp et al., 2005; Schaber et al., 2006; Zi et al., 2010), providing approaches on which we can now build further. Both pathways have previously been used in synthetic biology to demonstrate the feasibility of redirecting signal transduction (Park et al., 2003) as well as building artificial cell communication systems (Chen and Weiss, 2005).

In their work, Regot et al. (2011) have constructed and described 16 different types of engineered cells. In this study, we present kinetic models of four of these cells, which can be arranged in various combinations to perform five different logic operations (IDENTITY, NOT, OR, IMPLIES, NAND). All data necessary for model construction and parameterization have been obtained from previously published literature. The results from the work of Regot et al. are used only for model validation. The verified model is then employed for the identification of individual processes with highest impact on the functioning of the logic gates, for analyzing how culture density influences the system output and for determining the major sources of noise in the system. Finally it serves to propose how the current system could be transformed to operate on three discrete values.

## Materials and Methods

### Modeling sender and receiver cells

The sender cells respond to specific chemical input signals (salt, doxycycline, galactose) by producing the yeast pheromone alpha-factor, which is secreted into the culture medium and serves there as a signal for the receivers (reporter cells). The reporter cells contain the alpha-pheromone receptor, which activates the pheromone signaling pathway; the pathway is engineered to induce the expression of GFP (system output). Signaling and gene expression in all cells are described in our models with sets of ordinary differential equations (ODE). These are kept as simple as possible to prevent parameter overfitting and to reduce complexity of the final models. Table 1 contains an overview of the used data and references and presents the obtained parameter values. Initial concentrations for proteins are listed in Table 2. All species not listed in the table were initially set to 0. Yeast cell volume size has been set to 58 fL^1^ (Tyson et al., 1979; Jorgensen et al., 2002; Sherman, 2002; Tamaki et al., 2005), of which we assume the cytoplasm occupies 50% of volume (29 fL) and the nucleus 7% (4.06 fL; Biswas et al., 2003). Below, we explain the four different cell types used in this study.

**Table 1:** **Data used for model fitting and obtained parameter values**.

Cell	Reaction number	Parameter description	Parameter name	Estimated value	Experimental data	Reference
Salt-cell	–	Influence of NaCl concentration on osmostress strength	*w*	2.39	Fitted to time-course data (2 h) for Hog1 phosphorylation after stimulation of wild-type yeast cells with 0.4 and 0.8 M NaCl and to time-course data for internal glycerol after stimulation with 0.5 M NaCl	Macia et al. (2009), Klipp et al. (2005)
	r20	Activation of Pbs2	*k*_20_	758 ml/(mmol/s)		
	r21	Deactivation of Pbs2	*k*_21_	235 1/s		
	r22	Phosphorylation of Hog1	*k*_22_	113543 ml/(mmol/s)		
	r23	Cytoplasmic dephosphorylation of Hog1	*k*_23_	8.84 × 10^−5^ 1/s	
	r24	Nuclear dephosphorylation of Hog1	*k*_24_	0.0148 1/s	WT model verified with data for Hog1 phosphorylation after stimulation of wild-type yeast cells with 0.07, 0.1, 0.2, and 0.6 M NaCl	
	r25	Nuclear export of Hog1-PP	*k*_25_	34.5 1/s	
	r26	Nuclear import of Hog1-PP	*k*_26_	87.8 1/s	
	r27	Nuclear import of Hog1	*k*_27_	5.76 1/s	
	r28	Nuclear export of Hog1	*k*_28_	45.2 1/s	
	r29	Synthesis of internal osmolytes	*k*_29_	8170 1/s	
	r30	Leakage of internal osmolytes	*k*_30_	0.0035 1/s	Fitted to time-course data (50 min) for Hog1 phosphorylation after stimulation of *fps1*-Δ1 cells with 0.4 M NaCl^1^	Sergi Regot, personal communication
	–	Inhibition of internal osmolyte leakage by osmostress (through Fps1)	*Ki*_30_	100 ml/mmol		
	r31	Transcription from *PSTL1*	*k*_31_	1.5 × 10^−6^ 1/s	Fitted to time-course data for *STL1*-mRNA abundance after stimulation of wild-type yeast cells with 0.4 M NaCl^2^	Elzbieta Petelenz-Kurdziel, under review
Dox-cell	r38	Transcription from *PTet-Off*	*k*_38_	2 × 10^−12^ ml/(mmol*s)	Fitted to time-course data for the induction of a *Tet-Off* promoter in cells moved to medium without doxycycline, monitored by lacZ activity (compared to analogous data for *GAL1*-lacZ activity)	Gari et al. (1997) Johnston et al. (1994)
	r38		*Ki*_38_	1 × 10^7^ ml/mmol	Fitted to time-course data for *CLN1*-mRNA produced from a *Tet-Off* promoter after the addition of 1 μg/ml doxycycline	Gari et al. (1997)
					(Half-life for *CLN1*-mRNA ≈5 min)	Schneider et al. (2004)
Gal-cell	r37	Transcription from *PGAL1*	*k*_37_	1.2 × 10^−11^ 1/s	Fitted to time-course data for *GAL1*-mRNA abundance after shift of cells from glucose-to-galactose medium	Kundu et al. (2007)
					(Half-life for *GAL1*-mRNA ≈ 6 min)	Anderson and Parker (1998)
All sender cells	r31_deg	Degradation of MFalpha1-mRNA	*k*_31_deg_	0.00231 1/s	Half-life = 5 min	Herrick et al. (1990)
	r32	Pre-protein synthesis	*k*_32_	3 1/s	Average translation rate for yeast growing in rich medium, for an mRNA of 165 aa	von der Haar (2008)
	r33	Processing and export of alpha	*k*_33_	0.00315 1/s	Half-time = 3.67 min	Caplan et al. (1991)
Reporter cell	r1	Ste2-Alpha binding	*k*_1_	8 × 10^11^ ml/(mmol*s)	Fitted to dose-response curve for Fus3 phosphorylation and to dose-response curve for Ste2 receptor occupancy, both measured 15 min after stimulation of *bar1*Δ yeast cells with alpha-factor ranging from 0.01 to 100 nM	Yu et al. (2008)
	r2	Release of alpha from Ste2	*k*_2_	3250 1/s		
	r3	Ste2 synthesis^3^	v_Ste2_production_	6.95 × 10^−12^ mmol/(ml s)		
	r4	Ste2 degradation	*k*_4_	1.84 × 10^−5^ 1/s		
	r5	Ste2-Alpha degradation	*k*_5_	2.1 × 10^−5^ 1/s		
	r6	Activation of Ste5 complex	*k*_6_	18000 ml/(mmol*s)	
	r7	Inactivation of Ste5 complex	*k*_7_	0.0042 1/s	
	r8	Phosphorylation of Fus3	*k*_8_	3.2 × 10^10^ ml/(mmol*s)	
	r9	Cytoplasmic dephosphorylation of Fus3	*k*_9_	680 1/s	
	r10	Nuclear dephosphorylation of Fus3	*k*_10_	0.28 1/s	
	r12, r13	Nuclear import of Fus3 and Fus3PP^4^	*k*_nuc_imp_	16.8 1/s	
	r11, r14	Nuclear export of Fus3	*k*_nuc_exp_	85.7 1/s	
	r11	Relation of nuclear export of Fus3PP to export of Fus3^5^	*k*_small_	0.5	
	r34	Transcription from *PFUS1*	*k*_34_	4 × 10^−6^ 1/s	Fitted to time-course data for *FUS1*-mRNA abundance after stimulation of cells with 100 nM alpha-factor	Yu et al. (2008)
					(Half-life for *FUS1*-mRNA ≈3 min)	Herrick et al. (1990)
	r34_deg	Degradation of GFP-mRNA	*k*_34_deg_	0.00214 1/s	Half-life = 5.4 min	Hyde et al. (2002)
	r35	GFP synthesis	*k*_35_	2 1/s	Average translation rate for yeast growing in rich medium, for an mRNA of 238 aa	von der Haar (2008)
	r36	GFP folding and maturation	*k*_36_	9.625 × 10^−5^ 1/s	Maturation half-time = 120 min	Heim et al. (1995)

*^1^After fitting of the whole HOG module to data for wild-type cells, only parameters *k*_30_ and *Ki*_30_ were adjusted to fit data for the *fps1* −Δ 1 mutant*.

*^2^Maximal transcriptional induction was set to 10–15 mRNA molecules per cell for all modeled transcription reactions (von der Haar, 2008)*.

*^3^Parameter set in such a way that V_ste2_production_ = [ste2_0_]·*k*_4_, so if [Alpha_in_culture] = 0, then [Ste2_0] is the steady-state value*.

*^4^Parameters k_nuc_imp_ and k_nuc_exp_ are set in such a way that steady-state distribution of Fus3 between nucleus and cytoplasm is $\frac{\text{Fus}{\text{3}}_{\text{nuc}}}{\text{Fus}{\text{3}}_{\text{cyt}}}=1.4$ (Blackwell et al., 2003)*.

*^5^Parameter for nuclear export of Fus3PP is set at *k*_small_ ·*k*_nuc_exp_ to reflect more efficient nuclear retention of the phosphorylated species (van Drogen et al., 2001; Blackwell et al., 2003, 2007)*.

**Table 2:** **Initial concentrations used in the model (from Ghaemmaghami et al., 2003, except for Ste2 which is an average of published measurements taken from http://yeastpheromonemodel.org/wiki/Ste2_num)**.

Species	Initial concentration (nM)	Molecule number per cell
Pbs2	123.7	2160
Hog1n	340.5	832
Hog1c	340.5	5948
Ste2	378	6600
Inactive_Ste5complex^1^	38.5	672
Fus3n	568.4	1390
Fus3c	406	7090

*^1^Limiting factor is Ste7*.

#### Salt-cell

This cell (Cell#1 in Regot et al., 2011) responds to the presence of salt in the medium by producing the yeast pheromone alpha-factor; this is achieved by placing the alpha-factor encoding gene *MF*α*1* under the control of the *STL1* promoter, which is activated by the HOG MAPK signaling pathway. Additionally, the cells carry an *fps1*-Δ1 mutation, to prolong the response to osmostress (Tamás et al., 1999). We model the dynamics of this cell with a set of 12 equations, presented below. The first eight equations represent a highly simplified model of the HOG signaling pathway, inspired by the work of Zi et al. (2010). The model includes the following components: osmostress, the MAP kinase kinase (MAPKK) Pbs2, the MAP kinase Hog1, and internal osmolytes.

Osmostress depends on the presence of salt in the medium and internal osmolytes in the cytoplasm:

$$\begin{array}{rcll} & \left[\text{osmostress}\right] & & \\ & \;=\left\{\begin{array}{cc} w\left[\text{Salt}\right]-\left[\mathrm{Int}\text{\_}\text{osmo}\right],\; & w\left[\text{Salt}\right]-\left[\mathrm{Int}\text{\_}\text{osmo}\right]>0\\ 0,\; & \text{else}\\ \; \end{array}\right. & & \text{(1)} \end{array}$$

The signaling cascade is represented by Pbs2 and Hog1:

(2) $$\frac{\text{d[Pbs2]}}{\text{d}t}=-k_{20}\text{[Pbs2] [osmostress]+}k_{21}\text{[Pbs2pp]}$$

(3) $$\frac{\text{d[Pbs2pp]}}{\text{d}t}=k_{20}\text{[Pbs2] [osmostress]−}k_{21}\text{[Pbs2pp]}$$

The Hog1 MAPK is phosphorylated by active Pbs2 in the cytoplasm and dephosphorylated by phosphatases both in the cytoplasm and in the nucleus, to which it can be translocated with a rate that depends on its phosphorylation state:

(4) $$\begin{array}{l} \frac{\text{d[Hog1c]}}{\text{d}t}=-k_{22}\text{[Hog1c] [Pbs2pp]+}k_{23}\text{[Hog1ppc]}\\ \text{ –}k_{27}\text{[Hog1c]+}k_{28}[\text{Hog1n]}\frac{V_{\text{nucleus}}}{V_{\text{cytoplasm}}} \end{array}$$

(5) $$\begin{array}{l} \frac{\text{d[Hog1ppc]}}{\text{d}t}=k_{22}\text{[Hog1c] [Pbs2pp]−}k_{23}\text{[Hog1ppc]}\\ \text{ –}k_{26}\text{[Hog1ppc]+}k_{25}[\text{Hog1ppn]}\frac{V_{\text{nucleus}}}{V_{\text{cytoplasm}}} \end{array}$$

(6) $$\begin{array}{l} \frac{\text{d[Hog1n]}}{\text{d}t}=k_{24}\text{[Hog1ppn] +}k_{27}[\text{Hog1c]}\frac{V_{\text{cytoplasm}}}{V_{\text{nucleus}}}\\ \text{ –}k_{28}\text{[Hog1n]} \end{array}$$

(7) $$\begin{array}{l} \frac{\text{d[Hog1ppn]}}{\text{d}t}=-k_{24}\text{[Hog1ppn] +}k_{26}[\text{Hog1ppc]}\frac{V_{\text{cytoplasm}}}{V_{\text{nucleus}}}\\ \text{ –}k_{25}\text{[Hog1ppn]} \end{array}$$

Internal osmolytes are synthesized when active Hog1 is present in the cytoplasm but also leak from the cell with a rate dependent on osmostress (which represents regulation of the Fps1 glycerol channel by osmostress; Tamás et al., 1999):

(8) $$\frac{\text{d}\left[\text{Int\_osmo}\right]}{\text{d}t}=k_{29}\left[\text{Hog}1\text{ppc}\right]-\frac{k_{30}\left[\text{Int\_osmo}\right]}{1+Ki_{30}\left[\text{osmostress}\right]}$$

Alpha-factor expression is described by three ODEs representing mRNA synthesis and degradation, protein synthesis and degradation as well as protein processing and peptide export:

(9) $$\begin{array}{l} \frac{d\text{[MFalpha1\_mRNA]}}{dt}=k_{31}[\text{Hog1ppn]}\\ \text{ –}k_{31_{\deg}}\text{[MFalpha1\_mRNA]} \end{array}$$

(10) $$\begin{array}{l} \frac{\text{d[prepro\_Alpha]}}{\text{d}t}=k_{32}\text{[MFalpha1\_mRNA]}\\ \text{ –}k_{\text{33}}\text{ [prepro\_Alpha]} \end{array}$$

(11) $$\frac{\text{d [Alpha\_in\_medium]}}{\text{d}t}=\frac{k_{\text{33}}\text{ [prepro\_Alpha]}}{\text{dilution}}$$

where *dilution* is the factor by which the alpha-pheromone is diluted when exported from the cell cytoplasm to the culture medium; this factor decreases with time, as the cells in the culture divide and increase in number (assumed division time is 4 h, initial culture density is 5 × 10^6^ cells/ml for each cell type). We assume the following relation:

(12) $$\text{dilution}=13,800\cdot {\text{e}}^{-0.173\cdot \text{Time}\left[\text{h}\right]}$$

#### Dox-cell

The *dox-cell* (Cell#3; Regot et al., 2011) produces alpha-factor constitutively, from a Tet-Off promoter, and responds to the presence of doxycycline in the medium by turning off the expression of the *MFα1* gene. Since doxycycline enters the nucleus of yeast cells, no signal transduction is involved in this process and it is presented in the model by direct influence of the drug on the transcription reaction:

$$\begin{array}{rcll} \frac{\text{d}\left[\text{MFalpha}1\text{\_}\text{mRNA}\right]}{\text{d}t} & =\frac{k_{38}}{1+Ki_{38}\left[\text{DOX}\right]} & & \\ & \;-k_{31_{\deg}}\left[\text{MFalpha}1\text{\_}\text{mRNA}\right] & & \text{(13)} \end{array}$$

The two remaining equations of the alpha-factor expression module (Eqs 10 and 11) are the same as described above for the salt-cell model.

#### Gal-cell

This cell (Cell#5; Regot et al., 2011) carries the *MF*α*1* gene under the control of the *GAL1* promoter, such that it produces pheromone in response to the presence of galactose in the culture medium. Transcriptional induction is described by a single reaction:

(14) $$\frac{\text{d}\left[\text{MFalpha}1\text{\_}\text{mRNA}\right]}{\text{d}t}=k_{\text{Gal}}\left[\text{Gal}\right]-k_{31_{\deg}}\left[\text{MFalpha}1\text{\_}\text{mRNA}\right]$$

where

(15) $$k_{\text{Gal}}=\left\{\begin{array}{cc} 0,\; & \text{Time}<3\text{h}\\ {\text{k}}_{\text{37}},\; & \text{else}\\ \; \end{array}\right.$$

Here, we include a delay of 3 h in initiating the transcription of GAL genes after a shift from glucose- to galactose-containing medium (Li et al., 2000). For simulations where the cells are pre-cultured in galactose, no delay in GAL gene induction is included.

The two remaining equations of the alpha-factor expression module (Eqs 10 and 11) are the same as described above for the salt-cell model.

#### Reporter cell

The *reporter cell* (Cell#6 in Regot et al., 2011) is an alpha-factor responsive *MAT*a cell, which here carries additionally a *bar1*Δ deletion to enhance its pheromone sensitivity. The presence of pheromone causes activation of the Fus3 MAPK signaling pathway which leads to the induction of many genes, among them *FUS1*. In this cell, a GFP-encoding gene has been placed under the control of the *FUS1* promoter, causing the cell to produce GFP upon pheromone stimulation. GFP production by reporter cells is considered the output of the whole system.

Our highly simplified model of the pheromone pathway includes the Ste2 pheromone receptor, the Ste5 complex, which represents the kinase cascade, and the Fus3 MAPK which can be translocated to and from the nucleus in a manner dependent on its phosphorylation state. The pathway model consists of the following eight equations:

(16) $$\begin{array}{l} \frac{\text{d[Ste2]}}{\text{d}t}=v_{\text{Ste2\_production}}\text{ – }k_{\text{1}}\text{ [Ste2] [Alpha\_in\_medium]}\\ \text{ +}k_{\text{2}}\text{ [Ste2\_Ph] – }k_{\text{4}}\text{ [Ste2]} \end{array}$$

(17) $$\begin{array}{l} \frac{\text{d[Ste2\_Ph]}}{\text{d}t}=k_{\text{1}}\text{ [Ste2] [Alpha\_in\_medium] – }k_{\text{2}}\text{ [Ste2\_Ph]}\\ \text{ −}k_{\text{5}}\text{ [Ste2\_Ph] } \end{array}$$

(18) $$\begin{array}{l} \frac{\text{d[active\_Ste5complex]}}{\text{d}t}=k_{\text{6}}\text{ [inactive\_Ste5complex] [Ste2\_Ph]}\\ \text{ – }k_{\text{7}}\text{ [active\_Ste5complex] } \end{array}$$

(19) $$\begin{array}{l} \frac{\text{d[active\_Ste5complex]}}{\text{d}t}=k_{\text{6}}\text{ [inactive\_Ste5complex] }\\ \times \text{ [Ste2\_Ph] + }k_{\text{7}}\text{ [active\_Ste5complex] } \end{array}$$

(20) $$\begin{array}{l} \frac{\text{d[Fus3c]}}{\text{d}t}=\text{–}k_{\text{nuc\_imp}}\text{ [Fus3c] + }k_{\text{nuc\_exp}}\text{ [Fus3n]}\frac{V_{\text{nucleus}}}{V_{\text{cytoplasm}}}\\ \text{– }k_{\text{8}}\text{ [Fus3c] [active\_Ste5complex] + }k_{\text{9}}\text{ [Fus3ppc]} \end{array}$$

(21) $$\begin{array}{l} \frac{\text{d[Fus3c]}}{\text{d}t}=k_{\text{small}}\cdot \;k_{\text{nuc\_exp}}\text{ [Fus3ppn]}\frac{V_{\text{nucleus}}}{V_{\text{cytoplasm}}}\\ \text{ – }k_{\text{nuc\_imp}}\text{ [Fus3ppc] + }k_{\text{8}}\text{ [Fus3c] [active\_Ste5complex]}\\ \text{ –}k_{9}\text{[Fus3ppc]} \end{array}$$

(22) $$\begin{array}{l} \frac{\text{d[Fus3c]}}{\text{d}t}=\;k_{\text{nuc\_imp}}\text{ [Fus3c]}\frac{V_{\text{cytoplasm}}}{V_{\text{nucleus}}}-\;k_{\text{nuc\_exp}}[\text{Fus3n}]\\ \text{+}k_{10}\text{[Fus3ppc]} \end{array}$$

(23) $$\begin{array}{l} \frac{\text{d[Fus3ppn]}}{\text{d}t}=\text{ −}k_{\text{small}}\cdot \;k_{\text{nuc\_exp}}[\text{Fus3ppn}]\\ \text{+}k_{\text{nuc\_imp}}\text{[Fus3ppc]}\frac{V_{\text{cytoplasm}}}{V_{\text{nucleus}}}-\;k_{\text{10}}[\text{Fus3ppn}] \end{array}$$

Expression of GFP from the Fus3-responsive promoter is described by three ODEs representing mRNA synthesis and degradation, protein synthesis and degradation, and protein folding and maturation:

(24) $$\frac{\text{d[GFP\_mRNA]}}{\text{d}t}\text{=}k_{\text{34}}{\text{ [Fus3ppn] – k}}_{{\text{34}}_{\text{deg}}}\text{ [GFP\_mRNA]}$$

(25) $$\frac{\text{d[nascent\_GFP]}}{\text{d}t}\text{=}k_{\text{35}}\text{ [GFP\_mRNA] – }k_{36}\text{ [nascent\_GFP]}$$

(26) $$\frac{\text{d[mature\_GFP]}}{\text{d}t}\text{=}k_{36}\text{ [nascent\_GFP]}$$

### Deterministic simulations of logic gates

Parameter estimation and deterministic simulations were performed with the COPASI software (Hoops et al., 2006). Data used for parameter estimation is listed in Table 1.

### Stochastic simulations of reporter cells

For the analysis of noise in the system, the reporter cell model was simulated in a stochastic framework using CAIN^2^. All respective parameters were re-calculated from the deterministic model parameters and are listed in Table 3. For the initial assessment of noise generation by the reporter cell, we simulated the full stochastic model using the direct method of Gillespie (1976) and a quasi-steady-state-assumption (QSSA; Rao and Arkin, 2003) for the Ste2 receptor. The QSSA may be used for highly reactive species that follow a fast dynamics – these are rarely of interest and can be eliminated from the model, which leads to a shortening of the execution time. We generated 100 cell trajectories for different constant concentrations of alpha-factor (0.5, 2.5, 5 nM). For further stochastic simulations we modeled only the GFP expression module (transcription, translation, maturation). Here, we generated 1000 cell trajectories, also using the direct method, and using as input the time-dependent concentration of Fus3ppn obtained from deterministic simulations in Copasi. The deterministic result was first approximated by a polynomial function using the Curve Fitting Tool in Matlab 7.10.0 (Mathworks, Inc.). The derivative of this polynomial was then introduced into the CAIN model as the propensity of the synthesis of Fus3ppn. For the IDENTITY gate, where the first molecules of Fus3ppn appear in the deterministic model only after 400 s, the stochastic simulation was initiated at this time point.

**Table 3:** **Parameters for the CAIN model**.

Parameter	Value (1/s)
*k*_1_	45.82
*k*_2_	3250
*k*_3_	0.12144
*k*_4_	0.0000184
*k*_5_	0.000021
*k*_6_	0.000001031
*k*_7_	0.0042
*k*_8_	1.833
*k*_9_	680
*k*_10_	0.28
*k*_nuc_imp_	21.423
*k*_nuc_exp_	8.4
*k*_34_	0.0000286
*k*_34_deg_	0.00214
*k*_35_	2
*k*_36_	0.00009625

### Parameter sensitivity analysis

Parameter sensitivity analysis was conducted with Copasi (Hoops et al., 2006). The concentration of mature GFP was considered the relevant system output, and time-dependent sensitivity coefficients were calculated for the deterministic versions of the logic gates for nine time points selected from the 15- to 240-min range (every 15 or 30 min). The sensitivity coefficients are calculated by numerical differentiation using finite differences, with the delta parameter equal 10^−3^ and the minimal delta parameter equal 10^−12^. We analyzed in this manner all kinetic parameters, non-zero initial concentrations, the dilution factor, and the cell doubling time.

### Quantification of noise

Noise was quantified using CAIN. We calculated the variation coefficient from all 1000 generated trajectories for three selected time points: 1, 2, and 4 h. The variation coefficient was defined as the standard deviation normalized by the amount of molecules present at the analyzed time point.

## Results

### Modeling engineered logic-gate cells

**Figure 1:**
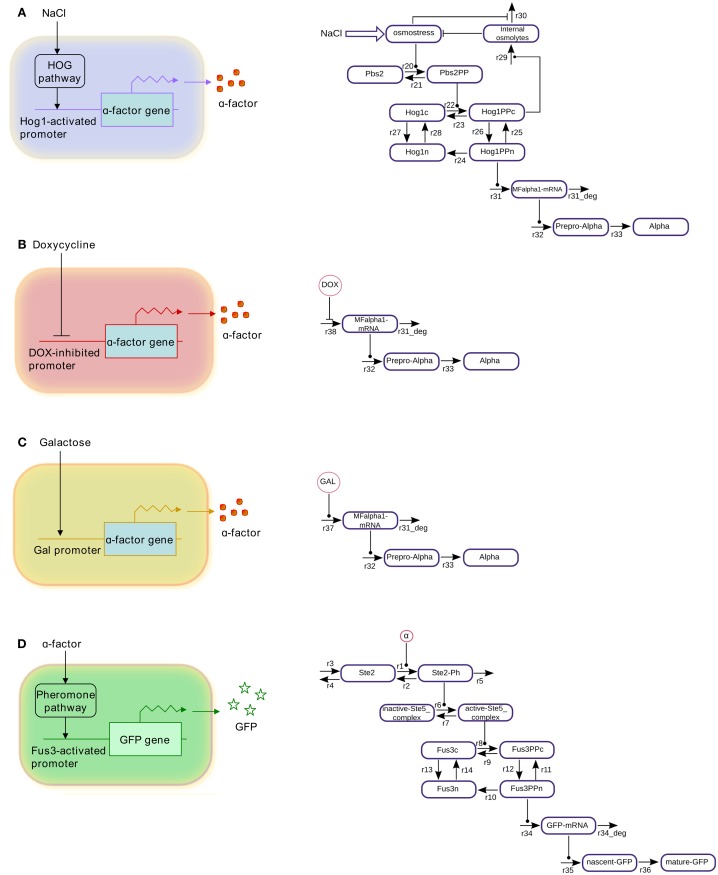
**Schematic representations and wiring graphs of the modeled cells**. **(A)** Salt-cell, **(B)** dox-cell, **(C)** gal-cell, **(D)** reporter cell.

**Figure 2:**
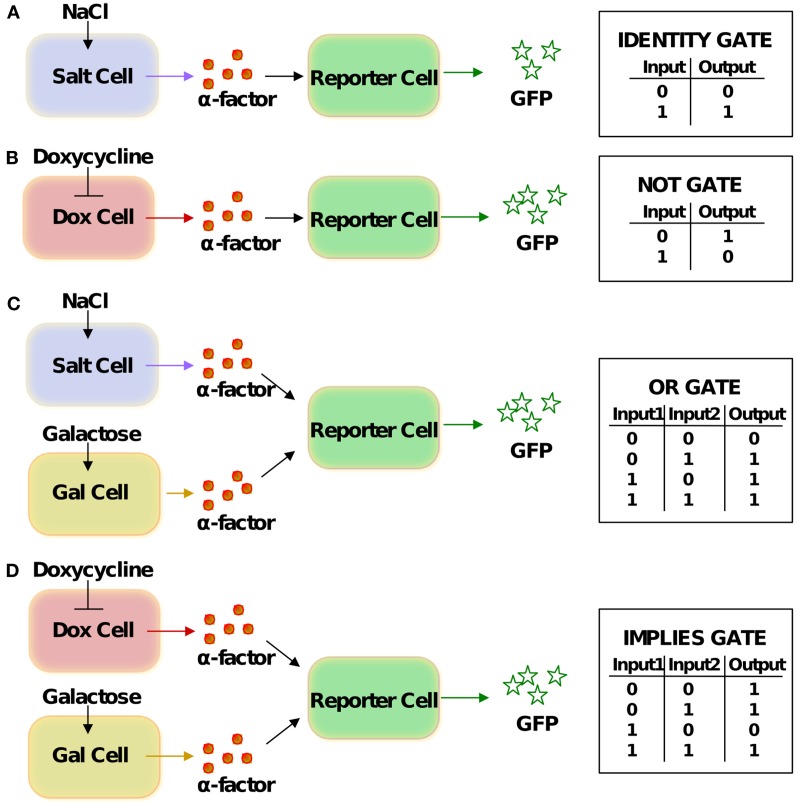
**Schemes of the logic gates and corresponding truth tables**. **(A)** IDENTITY gate, **(B)** NOT gate, **(C)** OR gate, **(D)** IMPLIES gate. The same cells can yield different logic functions by means of gate “reprogramming” (Regot et al., 2011): if glucose instead of galactose is viewed as one of the inputs for the gates in **(C,D)**, they perform the IMPLIES and NAND functions, respectively.

For simulating the behavior of the logic gates, we have first selected four different cell types from among the cells designed and engineered by Regot et al. (2011; schemes presenting the individual cells and corresponding wiring graphs are shown in Figure 1). These four cells can be combined in various ways, realizing as many as five different logic functions (Figure 2). We have then implemented mathematical models of these cells. By searching the available literature, we managed to assign approximate values to six of the total 37 kinetic parameters; the remaining parameters were fitted to published experimental time-course data collected from literature (see Table 1). Initial concentrations for all components were also taken from literature (Table 2). All the data used for logic-gate construction are related to original yeast molecules and pathways, not to the synthetically engineered cells. Data presented by Regot et al. were only used for comparison with the final models and for their validation (see below).

We consider in our analysis three different types of sender cells and one kind of reporter cell. The *salt-cell* uses the osmotic-stress-activated HOG signaling system to activate expression of alpha-factor upon stimulation with NaCl (Figure 1A). In the *dox-cell*, addition of doxycycline inhibits the Tet-Off promoter thereby switching off the expression of alpha-factor (Figure 1B). Finally, in the *gal-cell*, galactose activates the *GAL1* promoter to induce alpha-factor expression (Figure 1C). The *reporter cell* is in all cases an alpha-factor responsive *MAT*a cell, which has been engineered in such a way that the pheromone pathway activates expression of GFP (Figure 1D).

**Figure 3:**
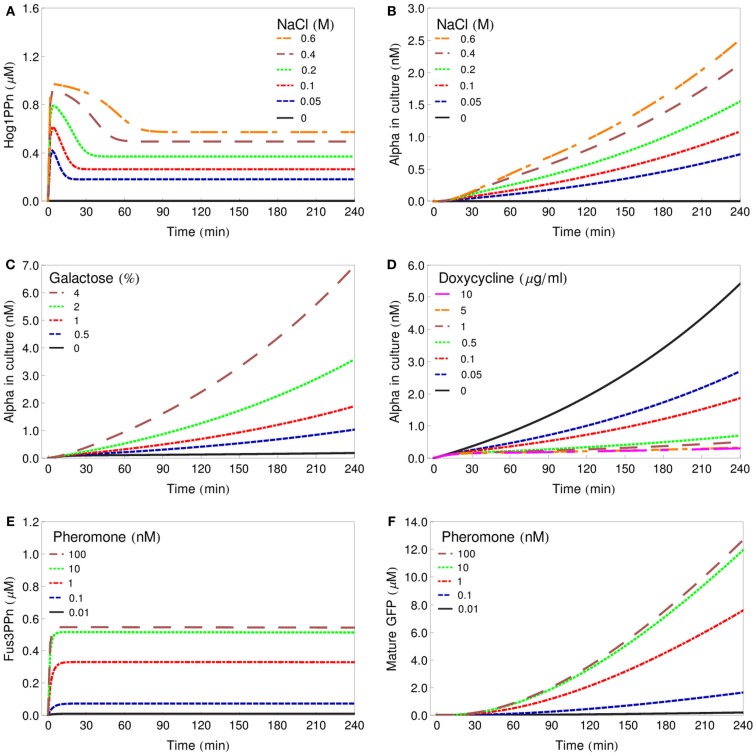
**Deterministic simulations of characteristic input/output relations for all model cells**. **(A)** Changes in Hog1ppn concentration after stimulation of the *salt-cell* with various concentrations of NaCl. **(B)** Alpha-factor secreted by the *salt-cell* after stimulation with various concentrations of NaCl. **(C)** Alpha-factor secreted by the *gal-cell* after stimulation with various concentrations of galactose. **(D)** Alpha-factor secreted by the *dox-cell* after treatment with various concentrations of doxycycline. **(E)** Changes in Fus3ppn concentration after stimulation of the reporter cell with various concentrations of alpha-factor. **(F)** Changes in the intracellular concentration of GFP in the reporter cell after stimulation with various concentrations of alpha-factor.

In order to represent the sender cells, we constructed minimalistic models of the HOG signaling system as well as of the GAL and DOX gene regulatory systems, showing only selected features. Transcription, translation, and export of alpha-factor are described by single reactions, respectively. The reporter cell model is composed of a simple model of the pheromone-response pathway and a GFP expression module, where transcription, translation, and GFP folding/maturation are also modeled by single reactions (for modeling details see Materials and Methods). Figure 3 presents simulations of characteristic input/output relations for all the individual cell models. The profiles of the phosphorylated (active) MAP kinases Hog1 and Fus3 represent the results of the parameter fitting procedures of the respective MAPK pathway modules. The profiles of alpha-factor generated by the sender cells and of GFP produced by the reporter cell represent model predictions. These species show a constant increase in concentration due to the fact that no degradation has been included into the models: we consider both spontaneous chemical degradation of alpha-factor in the culture medium and the degradation and bleaching of mature GFP to be slow processes, negligible on the time scale involved here (4–6 h of simulation).

### Deterministic simulations of logic functions

**Figure 4:**
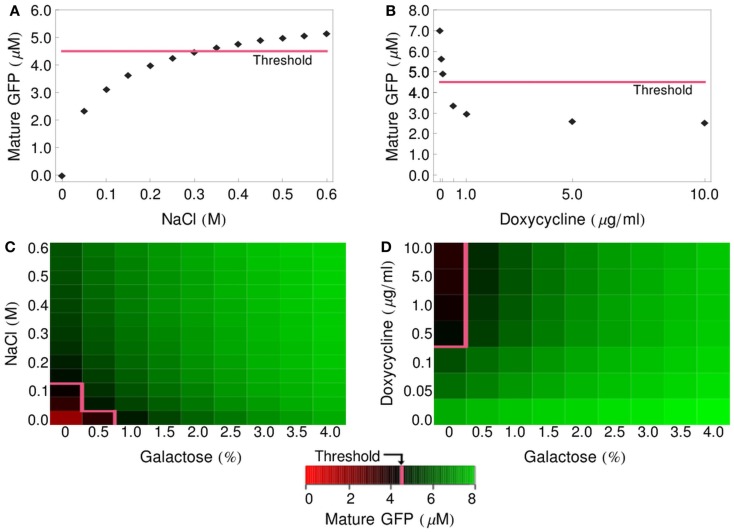
**Logic-gate output (GFP production) as a function of input concentration**. **(A)** GFP produced by the IDENTITY gate upon treatment with various concentrations of NaCl. **(B)** GFP produced by the NOT gate upon treatment with various concentrations of doxycycline. **(C)** Production of GFP by the OR gate upon treatment with various concentrations of NaCl and galactose. **(D)** Production of GFP by the IMPLIES gate upon treatment with various concentrations of doxycycline and galactose. On all graphs, the pink line depicts the threshold above which cells are scored as GFP-positive (4.5 μM GFP).

Having implemented and parameterized the cell models, we connected them into logic gates (Figure 2) and tested whether they faithfully represent the observed biological behavior. We performed deterministic simulations corresponding to the truth tables of four different logic functions: IDENTITY, NOT, OR, and IMPLIES (Figure 4). All four gates proved to function properly, generating biologically feasible cytoplasmic concentrations of GFP in the reporter cells and allowing us to define a common threshold for counting of a particular cell as GFP-positive, at the level of 4.5 μM cytoplasmic GFP. In all subsequent simulations, both deterministic and stochastic, we apply the 4.5-μM threshold for the interpretation of the logic results. We also postulate that in the *in vivo* implementation of Regot et al. (2011) the threshold must have been at a similar level.

In order to avoid the long induction times of the GAL genes that occur after a glucose-to-galactose shift (Li et al., 2000), Regot et al. (2011) pre-cultured the *gal-cells* overnight in galactose-containing medium. To reflect this for the simulations shown in Figure 4, we set the initial conditions of the *gal-cell* models to values obtained from a 16-h pre-simulation. These values are listed in Table 4. The pre-culturing leads to intracellular accumulation of mRNA for the *MFα1* gene and of unprocessed or partly processed alpha-factor polypeptides. At the time of mixing of the sender and receiver cells, the culture is put into fresh medium (containing appropriate input signals), so any alpha-factor that has been synthesized and excreted by the sender cells during the pre-culturing period is removed, but the intracellular species remain. A similar situation takes place for the *dox-cells*, which are pre-cultured in standard rich medium, without an addition of doxycycline, also resulting in an accumulation of alpha-factor mRNA and unprocessed polypeptide chains (values also listed in Table 4).

**Table 4:** **Initial conditions for *gal*-cells pre-cultured in the presence of 2% galactose and for *dox*-cells pre-cultured without doxycycline**.

Cell type and pre-culture conditions	Species	Initial concentration (nM)
Gal-cells pre-cultured overnight in medium with 2% galactose	MFalpha1-mRNA	0.571
	Prepro-alpha	544
Dox-cells pre-cultured overnight in medium without doxycycline	MFalpha1-mRNA	0.866
	Prepro-alpha	825

These pre-accumulated species are the source of alpha-factor synthesis and export even under conditions that block new transcription of the *MFα1* gene (glucose shift of *gal-cells*, addition of doxycycline for *dox-cells*). This effect is clearly visible on the graphs presented in Figure 4. In gates involving the *gal-* and *dox-cells* (NOT, OR, IMPLIES; Figures 4B–D, respectively) even unstimulated cells still produce significant amounts of alpha-factor and, consequently, GFP: in the NOT gate, even a large dose of doxycycline does not prevent the accumulation of over 2 μM GFP. In the OR and IMPLIES gates this effect leads to the extremely narrow ranges of concentrations that ensure a negative output (see pink threshold line).

**Figure 5:**
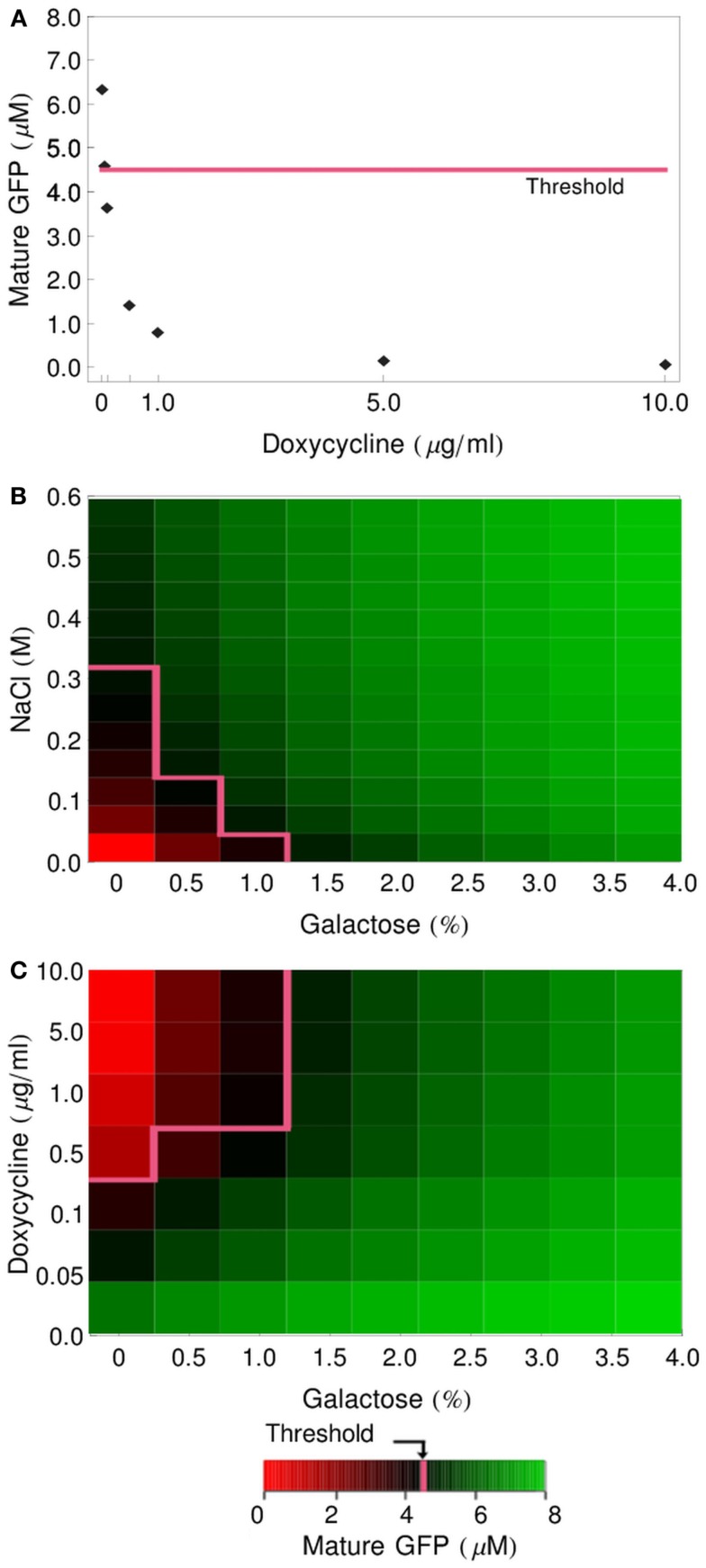
**Influence of pre-accumulation of alpha-factor mRNA and precursors in *gal-* and *dox-cells* on gate functioning**. Initial concentrations of MFalpha1-mRNA and prepro-Alpha in *gal*- and *dox-cells* were set to 0 and simulations were performed as in Figure 4. **(A)** GFP produced by the NOT gate. **(B)** GFP production in the OR gate. **(C)** GFP production in the IMPLIES gate. The pink line depicts the GFP threshold of 4.5 μM.

The half-lives of MFalpha1-mRNA and prepro-Alpha are short in our model (5 and 3.67 min, respectively; Table 1), so if any delay in the handling of the cells occurred – if the spinning down, mixing with reporter cells in fresh medium and adding of input signals took, e.g., 15 min – the amount of pre-accumulated mRNA and polypeptides would decrease significantly. To test how strong the influence of these pre-accumulated species on the functioning of the gates is, we performed simulations where we set the initial concentrations for these species back to zero. The graphs presented in Figure 5 show that the modified gates are indeed prevented from generating any GFP when not stimulated.

### Parameter sensitivity analysis

To determine the influence of individual parameters on the output of the system – the amount of GFP produced by the reporter cells during 4 h of incubation of the IDENTITY gate with 0.4 M NaCl – we performed a parameter sensitivity analysis. We calculated time-dependent sensitivity coefficients for selected time points in the 15- to 240-min range (see Materials and Methods). Sensitivity coefficients are a measure of the influence of an infinitesimally small change in the analyzed parameter on the concentration of mature GFP at a given time point. We calculated sensitivity coefficients in respect to all kinetic parameters, non-zero initial concentrations, the dilution factor, and cell doubling time.

**Figure 6:**
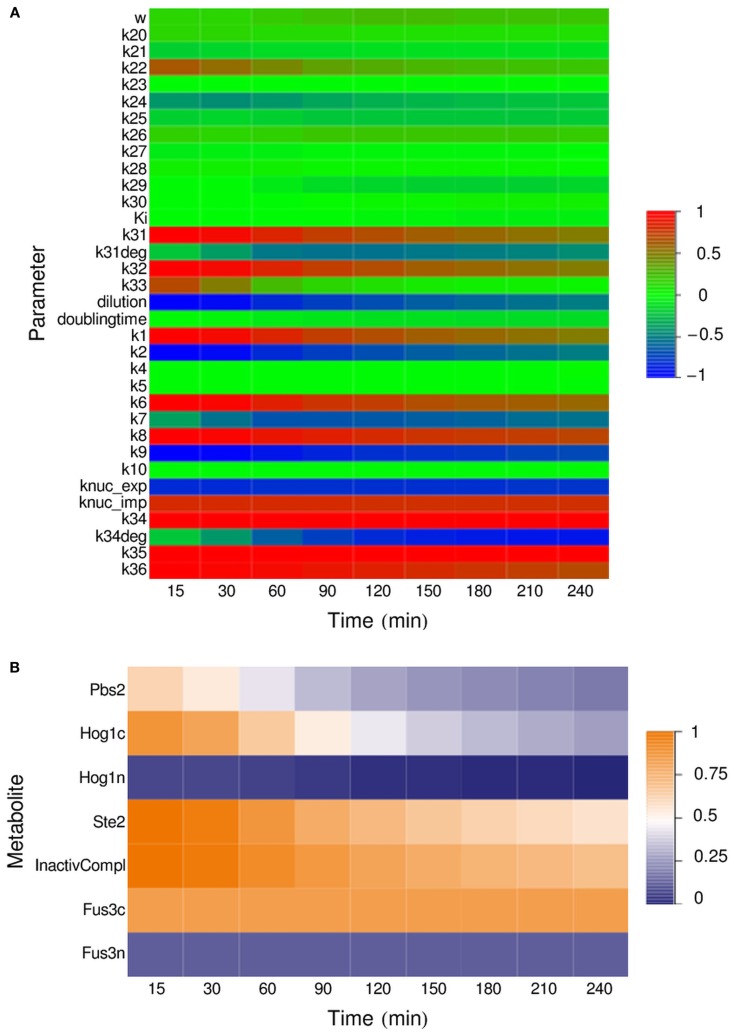
**Parameter sensitivity analysis for the IDENTITY gate stimulated with 0.4 M salt**. All kinetic parameters, dilution factor, and cell doubling time **(A)** as well as all non-zero initial concentrations **(B)** were varied and their relative influence on system output at the indicated time points was determined (for details, see Materials and Methods).

The results, presented in Figure 6, show that the HOG module parameters have very little influence on the final output – only the initial amount of cytoplasmic Hog1 and the rate of its phosphorylation have an effect. In the pheromone pathway, there are three key reactions – the binding of alpha-factor to its receptor, the activation of the Ste5 complex and of Fus3 itself, and the nuclear shuttling of Fus3 – where the kinetic parameters and the initial concentrations of the substrates have significant impact on the output. The initial amount of non-phosphorylated MAP kinases in the nucleus has very little influence, for both Hog1 and Fus3. This is likely due to the fact that the number of MAPK molecules in the nuclei is initially low and their transport to the cytoplasm – where MAPK activation takes place – has a relatively small impact on cytoplasmic kinase concentration. Most interesting, however, are the results for the two protein production modules: the parameters for transcription and translation of the alpha-factor gene are the key parameters in the sender cell, and the module for transcription and synthesis of GFP by the reporter cell seems to be the crucial part of the system.

The sensitivity analysis also revealed that the initial density of the cell cultures – described by the dilution factor – has strong influence on gate behavior (Figure 6A). This is because the amount of alpha-factor released into a volume unit is directly proportional to the number of sender cells per volume unit, and alpha-factor concentration influences the rate of its own binding to the Ste2 receptor – one of the key reactions in the reporter cell (see above). On the other hand, the cell doubling time, which we have arbitrarily set to 4 h, turned out to have little impact on system output, despite the obvious connection between doubling time and culture density. We thus investigated this phenomenon further.

### Influence of culture density and doubling time on gate functioning

**Figure 7:**
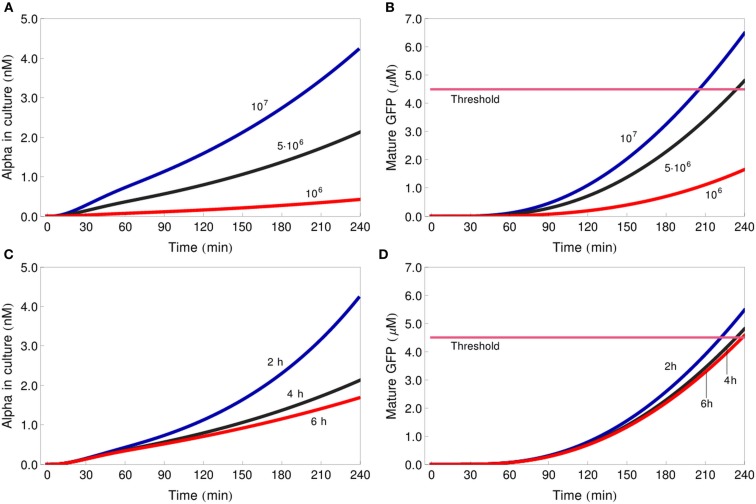
**Influence of dilution factor and cell doubling time on gate functioning**. Changes in concentration of alpha-factor in the culture medium **(A,C)** and of mature GFP in the reporter cells **(B,D)** for three different initial culture densities: 10^6^ cells/ml (red line), 5 × 10^6^ cells/ml (black line), 10^7^ cells/ml [blue line **(A,B)**] and three different cell doubling times: 6 h (red line), 4 h (black line), 2 h (blue line; **C,D)**. All simulations represent the IDENTITY gate treated with 0.4 M NaCl. The pink line depicts the GFP-concentration threshold.

The parameter sensitivity analysis identified the dilution factor, but not the cell division rate, as one of the key parameters that determine the outcome of the system. To better understand this result, we analyzed the influence of culture density on gate functioning in more detail. We first simulated the behavior of the IDENTITY gate assuming that the initial culture concentration were five times lower (10^6^ cells/ml) or two times higher (10^7^ cells/ml) than the standard concentration used in our previous simulations (5 × 10^6^ cells/ml). Next, we changed only the doubling time: from the previously used 4–2 or 6 h. On the graphs presented in Figure 7 we see that if the initial culture density is modified, the curves for secreted alpha-factor immediately acquire different slopes (Figure 7A), whereas manipulating cell growth rate differentiates the slopes only after about 120 min of incubation (Figure 7C). In the first case, differences in alpha-factor concentration lead to large differences in GFP production (Figure 7B). In the second case, although the final alpha-factor concentrations are also far apart (e.g., compare curves for doubling times of 2 and 4 h at the 240-min time point in Figure 7C), the 4-h incubation period of the experiment is too short for these differences to result in large differences on the GFP-production level (Figure 7D). If the gates were incubated for significantly longer time periods, the cell doubling time would gain high impact on GFP levels, but if the 4-h incubation period is kept, changing the initial number of cells is a much more efficient way of influencing system output.

### Stochastic simulations of logic functions

Since Regot et al. (2011) have presented in their paper single-cell data for the logic-gate system, the deterministic model simulations presented above were not fully appropriate for the comparison of our results with their data. Thus, in order to verify our models, we decided to conduct stochastic simulations that would predict the behavior of whole populations of cells.

We needed predictions showing the behavior of the reporter cells in each culture, i.e., showing how many GFP-positive cells are present in the culture at given time. Since the alpha-factor produced by the sender cells is secreted into the culture medium and cultures are well mixed (kept in shaking flasks), at this stage there was no need for simulating single-cell data: only the global concentration of alpha-factor in the whole culture was important. For this reason, we only implemented the reporter cell model in a stochastic framework, using CAIN (see text footnote 2 for modeling details see Materials and Methods). We then analyzed its behavior in response to stimulation with constant concentrations of alpha-factor. In order to reduce model complexity, we used a steady-state approximation for the number of ligand-bound Ste2 receptor molecules. We generated 100 individual cell trajectories for alpha-factor inputs of 0.5, 2.5, and 5 nM. This allowed us to estimate the amount of noise associated with various parts of the model. The results indicate that the pathway module generates cell-to-cell variation of about 3–8% at the level of active Fus3 MAP kinase concentration, while the whole model reaches variation of 7–23% at the level of GFP production (Table 5). After 1 h of simulation, the GFP module is responsible for 70–80% of total variation, but after 4 h for only 25–30%. This is due to the fact that – for constant alpha-factor inputs – the relative variation of GFP decreases with time because the number of GFP molecules increases. On the contrary, for Fus3ppn this is not the case, because at the 1-h time point its molecule number has already reached a stable value (Figure 3E) and so also cell-to-cell variation remains stable throughout the analyzed time range. Consistent with the larger number of molecules involved in all reactions after stimulation with higher pheromone, the amount of noise at both levels decreases with increasing alpha-factor concentration.

**Table 5:** **Quantification of cell-to-cell variation in the logic-gate cultures**.

Model	Input		Input concentration		Coefficient of variation for Fus3PPn (%)			Coefficient of variation for mature GFP (%)		
					1 h	2 h	4 h	1 h	2 h	4 h
Full model of reporter cell	Alpha-factor (nM)		0.5		7.64	7.72	7.65	22.88	16.32	10.33
			2.5		4.25	4.37	3.58	14.71	10.99	7.87
			5		3.35	3.73	3.68	15.99	11.62	7.17
IDENTITY gate	NaCl (M)		0.0		0.00	0.00	0.00	0.00	0.00	0.00
			0.1		6.34	4.62	3.55	47.50	24.00	12.56
			0.2		5.36	4.13	3.27	41.16	20.08	11.19
			0.3		4.90	3.79	3.14	38.90	19.47	10.64
			0.4		4.82	3.79	3.10	37.66	19.06	10.62
			0.5		4.52	3.60	3.15	34.68	17.92	10.27
			0.6		4.38	3.56	3.09	34.35	18.15	9.93
NOT gate	DOX (μg/ml)		0.0		3.63	3.20	2.86	26.39	14.59	8.51
			0.05		4.29	3.52	3.05	28.91	16.51	9.78
			0.1		4.58	3.84	3.19	28.86	16.85	10.27
			0.5		5.49	4.79	3.94	32.89	20.72	12.89
			1.0		5.93	5.23	4.24	34.34	21.65	13.30
			5.0		5.81	5.37	4.85	34.05	22.45	14.35
			10.0		6.05	5.61	4.89	35.20	22.52	14.83
OR gate	NaCl (M)	GAL (%)	0.0	0	7.16	6.69	5.92	41.45	26.83	17.78
			0.4	0	4.15	3.41	2.99	30.01	16.37	9.58
			0.0	2	4.20	3.40	2.99	30.12	16.09	9.54
			0.4	2	3.68	3.10	2.83	25.15	14.16	8.56
IMPLIES gate	DOX (μg/ml)	GAL (%)	0.0	0	3.54	3.18	2.87	23.20	14.55	9.21
			0.0	2	3.29	2.98	2.77	21.64	13.37	8.76
			10.0	0	4.74	4.56	3.94	27.60	18.51	12.45
			10.0	2	3.88	3.36	2.94	23.87	14.05	8.91

**Figure 8:**
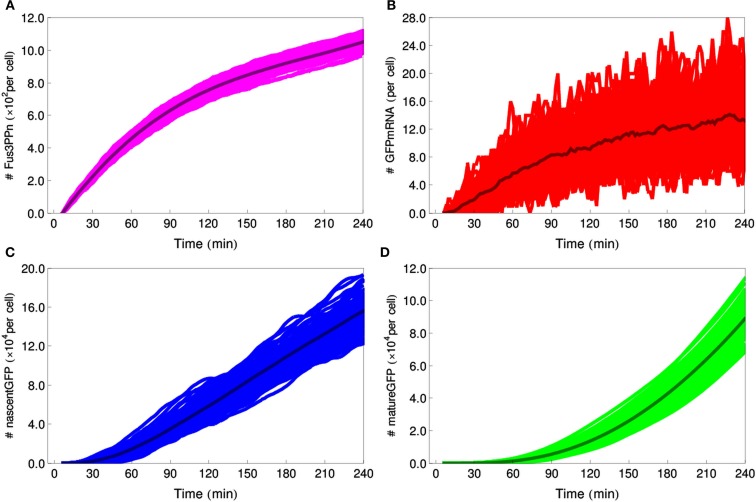
**Stochastic simulations of the IDENTITY gate stimulated with 0.4 M salt**. Hundred individual cell trajectories are shown for each selected species: **(A)** number of Fus3ppn molecules per cell, **(B)** GFP-mRNA molecules, **(C)** nascent GFP polypeptides, **(D)** mature GFP molecules.

The initial simulations of the stochastic reporter cell model have made it clear that the model requires too much computational power to be used in logic-gate simulations, where the alpha-factor concentration changes during the simulation time and no steady-state approximation can be applied. We decided therefore to modify our approach. We used the deterministic reporter cell model to generate curves describing how the mean concentration of Fus3ppn changes over time, and we used these curves to derive reaction propensities that were used as input for the stochastic model, which now encompassed only Fus3ppn and the GFP-production module (for details see Materials and Methods). The amount of noise associated with cell-to-cell variation on the Fus3ppn level that has been obtained from this procedure was in the same range of values as that produced by the full model (3–5% for alpha-factor concentrations rising from 0 to 2.5 nM during the simulation; Table 5 and Figure 8, compare with alpha-factor concentrations in Figure 3B) and was thus considered a satisfying approximation. We applied this procedure in all further stochastic simulations.

**Figure 9:**
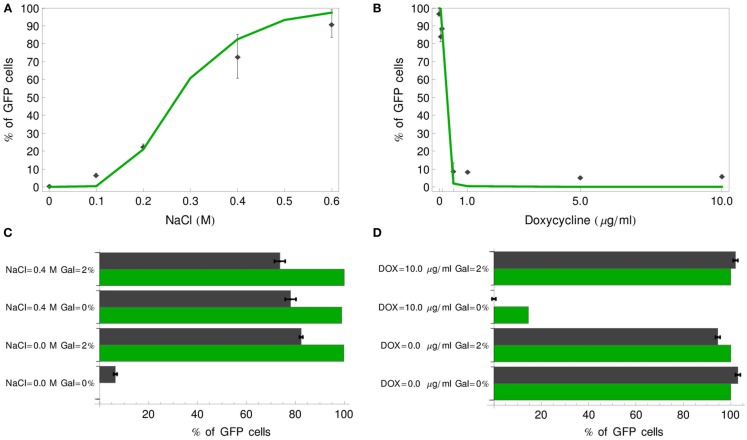
**Comparison of stochastic simulation results with experimental data of Regot et al. (2011)**. Percentage of GFP-positive cells (green line) is plotted for a range of input concentrations for the IDENTITY **(A)** and NOT **(B)** gates and compared with experimental results (black diamonds). For the OR **(C)** and IMPLIES **(D)** gates four input concentration combinations were tested and the simulation results are plotted as green bars, whereas black bars correspond to the experimental data.

Having established the stochastic model, we first verified whether our models reproduce the experimental results of Regot et al. (2011), obtained by fluorescence activated cell sorting (FACS). We performed simulations corresponding to experiments with various concentrations of inputs for the single-input gates (IDENTITY and NOT) and with four concentration combinations for the double-input gates (OR and IMPLIES). All cells reaching a GFP concentration of 4.5 μM or more were scored as positive. For each simulation, we generated 1000 individual cell trajectories. In all cases, our models proved slightly more digital than the experimental gates (Figure 9). For most concentrations these differences are minor. The largest discrepancies are visible for the OR gate, where the model predicts either 0 or 100% of positive cells, while the experiment yielded ca. 7% positive cells for the negative gate output and 75–80% positive cells for positive gate output, and for the negative output of the IMPLIES gate, where the model predicts more than 10% positive cells, while the experiment yielded none. These minor deviations are likely due to a cumulative effect of several issues: (i) some ambiguity in our setting of the GFP threshold at 4.5 μM (Figure 4); (ii) potential decline of pre-accumulated mRNA and precursors during the slightly variable period necessary for moving cells from pre-cultures to fresh medium, an effect that has been already discussed for the deterministic gates (Figures 4 and 5); (iii) inaccuracy resulting from our method of approximating cell-to-cell variation at the Fus3ppn level (Table 5). Still, taking into account that the models were built from literature data, without the use of any quantitative data about the engineered cells, we consider that the model predictions fit the experimental data well.

We also quantified the noise generated by the stochastic models of all four logic gates (Table 5). Again, as mentioned above for the reporter cell model, system noise decreases with increasing alpha-factor production by sender cells due to higher numbers of molecules involved in the reactions. But, in contrast to the results obtained for constant alpha-factor inputs, here both Fus3ppn and GFP molecule numbers rise throughout the simulation time and, therefore, the corresponding variation coefficients decrease. In these models, the GFP-production module is consistently responsible for approximately 65–70% of total system noise at the end of the simulations and it is clearly the main source of noise in the system.

### System modification: Three-value logic functions

One of the advantages of the logic-gates system developed by Regot et al. (2011) is its plasticity. This includes, e.g., logic reprogramming, described by the authors of the discussed paper (see also legend for Figure 2). However, many other ways of modifying the system could be suggested. Adding one new cell type to the cultures can change the output with relatively little experimental effort. As an example of a novel system modification, we present in this section a means of transforming the classic 0–1 identity gate into a three-value identity function, with three possible inputs: 0, 0.1, 0.4 M NaCl, and three respective outputs: no GFP-positive cells, one fluorescence-positive population, or two fluorescent populations.

**Figure 10:**
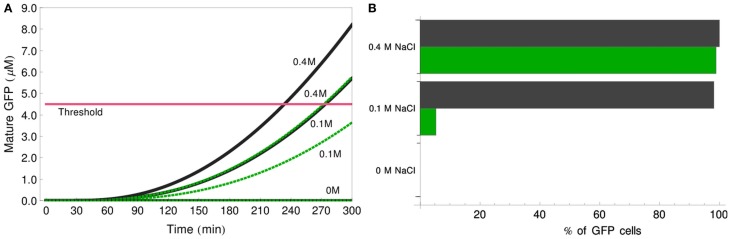
**Three-value IDENTITY gate**. **(A)** Deterministic simulation showing the amount of GFP produced by each of the two reporter cell populations, for stimulation with 0, 0.1, or 0.4 M NaCl. Black lines – reporter cells with wild-type Ste2, green dotted lines – reporters carrying Ste2^F262A^. The pink line depicts the 4.5-μM GFP threshold. **(B)** Results of stochastic simulation showing percentage of reporter cells of each type that score fluorescence-positive after stimulation with 0, 0.1, or 0.4 M NaCl. Black bars – reporter cells with wild-type Ste2, green bars – reporters carrying Ste2^F262A^.

Adding a new reporter cell population to the original IDENTITY gate could realize the implementation of such a function. In our theoretical example, we create a new type of reporter cells by substituting in these cells the wild-type Ste2-alpha-factor receptor protein with a mutated version, Ste2^F262A^. This mutant protein has two times lower ligand affinity than its wild-type counterpart, but it leaves the signaling competence of the cells unaffected (Lee et al., 2002). We added to our models of the IDENTITY gate this third population of cells, differing from the original reporter cells only by the value of parameter *k*_1_ which is here equal to 4 × 10^11^ ml/(mmol s). We simulated the behavior of this modified gate in response to treatment with 0, 0.1, or 0.4 M salt (Figure 10). The deterministic simulations in Figure 10A show that the responses of the mutant reporter cells are significantly slower than that of the original reporter cells. This allows for differentiation between the response to low (0.1 M) and high (0.4 M) salt. We find that good differentiation between these two situations is achieved after 5 h of incubation. At this stage, the original reporter cells exceed the threshold both after low- and high-salt treatment, whereas the Ste2-mutant cells score as GFP-positive only in high salt. Figure 10B shows the results of stochastic simulations: if no salt is added, no GFP-positive cells appear in the culture, if 0.1 M salt is added, 50% of the reporter cells (1/3 of all cells in the culture) become fluorescent, but if 0.4 M salt is added, almost 100% of the reporter cells (2/3 of all cells) respond. These last two situations can be discriminated in a FACS analysis either simply by the relative number of cells shifting to higher GFP-fluorescence or by the appearance of fluorescent populations in two color channels if a different fluorophore is used for one of the reporter cell types. This simple example demonstrates how easily the multicellular gate system can be modified to perform novel tasks.

## Discussion

Predicting the behavior of an artificial biological device is one of the challenges of synthetic biology. In this work we set out to show that a kinetic mathematical model built from dispersed literature data can yield useful predictions about the functioning of a synthetic biological system. We present here kinetic models of a published logic-gates system (Regot et al., 2011) and we show that the model simulations provide good predictions when compared to published experimental data.

Our models perform well despite a number of simplifying assumptions. In order to reduce model complexity, many known components and some regulatory interactions were left out of the model structures – this applies to all modeled parts: the MAP kinase pathways, the galactose-, and doxycycline-regulated transcriptional modules, as well as the synthesis and transport of pheromone and GFP in the cells. It is important to note here that this was facilitated by the fact that these subsystems have been extensively characterized in the literature, not only experimentally but also through previous modeling attempts. This provided us both with experimental data for validation of the individual modules (Table 1) and with tested, simple models that could serve as starting points for modification according to our needs (e.g., Li et al., 2000; Kofahl and Klipp, 2004; Klipp et al., 2005; Schaber et al., 2006; Zi et al., 2010). It is commonly postulated that a library of standardized, experimentally characterized, genetic parts is necessary for full development of the synthetic biology field (Canton et al., 2008). Here, we argue – along with others (Marchisio and Stelling, 2009; Matsuoka et al., 2009; Cooling et al., 2010) – that standard parts should be accompanied by a set of published kinetic models that would facilitate model-guided biological design.

We applied more simplifications in our modeling approach. We used a global translation rate – calculated for logarithmically growing yeast cells in rich glucose medium (von der Haar, 2008) – for all translation events, neglecting differences both between individual transcripts and between different growth conditions, such as carbon source (glucose or galactose) or applied stress (high salt). Cell doubling time is also assumed to be the same for all simulated cells, independent of growth conditions. Since all the available kinetic transcription rate data is relative, we needed to make assumptions about the number of mRNA molecules corresponding to maximal induction of the modeled transcripts, and again we used a common value for all transcripts, based on general information about yeast cells (von der Haar, 2008). Similarly, the available kinetic data for MAP kinase activation is also relative and requires assumptions concerning maximal activity – here, we assumed that full activation under standard stimulation conditions (100 nM alpha-factor and 0.4 M NaCl, respectively) means that 50% of all available Fus3 and Hog1 molecules become phosphorylated. All these assumptions need to be kept in mind when the models are used and interpreted – e.g., the generalized cell doubling time remains an acceptable simplification as long as the simulation times do not exceed a few hours.

The presented models have given us much additional insight into the analyzed system. Parameter sensitivity analysis allowed us to determine which parameters should be manipulated to most efficiently influence system output. The best way to increase the amount of GFP produced during 4 h of incubation is by increasing the rate of GFP transcription or translation or by increasing the half-life of the GFP-mRNA (Figure 6). Experimentally, this information could be very useful to define feasible scenarios, such as (i) selection of a different Fus3-dependent promoter for expression of the fluorescent protein, (ii) introduction of a second copy of the expression construct, or (iii) artificial stabilization of the corresponding mRNA. Choosing a fluorescent protein with faster maturation properties – an intuitive way of increasing fluorescence production – turns out to be less efficient. Our analysis also points to another, even more simple way of manipulating system output: by changing the number of cells used for the experiment (Figure 7). Although this method is less efficient than changes in the GFP-production module (Figure 6), it is by far the easiest and it could prove useful.

Finally, in the last section we show how the system could be modified to perform new functions. We present a modification that transforms the classic IDENTITY gate into a three-value (0-1-2) logic function. We would like to stress here how easy it is to test a novel design concept when a mathematical model is available. We were able to quickly and cheaply analyze the effects of several different *STE2* mutations that are described in the literature (results not shown) and to identify a mutant that allowed us to obtain the desired effect. Obviously, experimental testing of this concept would have cost much more time and money.

## Conflict of Interest Statement

The authors declare that the research was conducted in the absence of any commercial or financial relationships that could be construed as a potential conflict of interest.

## Supplemental Material

The Supplementary Material for this article can be found online at: http://www.frontiersin.org/Systems_Physiology/10.3389/fphys.2012.00287/abstract

File S1 **Copasi file (.cps) of IDENTITY gate model**.

File S2 **Copasi file (.cps) of NOT gate model**.

File S3 **Copasi file (.cps) of OR gate model**.

File S4 **Copasi file (.cps) of IMPLIES gate model**.

File S5 **CAIN model (.xml) of reporter cell**.

## Footnotes

^1^http://yeastpheromonemodel.org/wiki/Cell_volume

^2^http://cain.sourceforge.net/

## Research Topic:

From structural to molecular systems biology: experimental and computational approaches to unravel mechanisms of kinase activity regulation in cancer and neurodegeneration

## Topic Editor:

Matteo Barberis, Humboldt University Berlin, Germany; Max Planck Institute for Molecular Genetics, Berlin, Germany
